# Allium sativum extract as an irrigant in pulpectomy of primary molars: A 12‐month short‐term evaluation

**DOI:** 10.1002/cre2.197

**Published:** 2019-06-26

**Authors:** Ahmad Abdel Hamid Elheeny

**Affiliations:** ^1^ Faculty of Dentistry Minia University Minya Egypt

**Keywords:** garlic extract, primary molars, pulpectomy, sodium hypochlorite

## Abstract

This study indented to assess the clinical and radiographic assessment of Allium sativum extract as an intracanal irrigant for pulpectomy of primary molars. Ninety children with 110 teeth submitted were categorized into two groups. Clinical and radiographic success rates were checked at 3, 6, and 12 months. Qui‐square test at a level of significance was ˂0.05. There was no statistically significant difference (*p* ˂ .05) between the two groups that has not been detected clinically or radiographically. Clinical and radiographic success rates of garlic extract at 3 months were (80% and 72.7%), which declined at 6 and 12 months to be 76.4% 6 and 74.5% respectively. For NaOCl group, clinical and radiographic success rates were 87.3% and 85.5% at 3 months, 87.3% and 87.3% at 6 months and 89.1% and 87.3% at 12 months. A. sativum extract can be used efficiently as an irrigant for pulpectomy of primary molar root canals.

## 1 INTRODUCTION

Deciduous tooth preservation near to their shedding time is a vital issue from several aspects such as the child's growth and development, esthetic, functional, psychological, and dental arch integrity aspects (Barberia, Lucavechi, Cardenas, & Maroto, 2006; Setia, Pandit, Srivastava, Gugnani, & Sekhon, 2013; Tunison, Flores‐Mir, ElBadrawy, Nassar, & El‐Bialy, 2008). Pulpectomy is one of treatment choices to achieve this goal and it is suitable for treatment of primary teeth with irreversible pulpitis, necrosis, or periodontitis due to caries or trauma (Barcelos, Tannure, Gleiser, Luiz, & Primo, 2012; Brustolin, Mariath, Ardenghi, & Casagrande, 2017). The complex nature of the deciduous root canal system facilitates the spread of pathogenic microbes through lateral and accessory canals, dentinal tubules, apical ramifications, and the possibility of harming the tooth germ of permanent successor, and subsequently, these barriers make total removal of necrotic tissue by instrumentation alone impossible (Camp, 2008; Pazelli et al., 2003; Pozos‐Guillen, Garcia‐Flores, Esparza‐Villalpando, & Garrocho‐Rangel, 2016; Vianna et al., 2004). Therefore, irrigation is a replenishing step to mechanical instrumentation during root canal preparation to create a disinfectant atmosphere inside the root canal (Prabhakaran & Mariswamy, 2018). Another critical function of intracanal irrigation is elimination of the smear layer that raises dentin permeability and allows permeation of intracanal medication (Pashley, Michelich, & Kehl, 1981; Teixeira, Felippe, & Felippe, 2005; Zehnder, 2006). Sodium hypochlorite (NaOCl) is a frequently used root canal irrigant used by dentists (Hulsmann & Hahn, 2000). Although NaOCl is capable of dissolving tissues and has potent antibacterial effect (Dube & Jain, 2018; da Silva, Alves, Lutterbach, Paiva, & Ferreira, 2018; Rocas & Siqueira, 2011), it has several demerits including cytotoxic effect, especially when introduced into the periapical region (Fouad, 2011; Hulsmann & Hahn, 2000; Kumar et al., 2018), accidental injuries to the eyes, skin, and mucous membrane (Serper, Ozbek, & Calt, 2004; Singh, 2010) and allergic reaction is also reported (Baser Can, Karapinar Kazandag, & Kaptan, 2015). Also, it can damage permanent tooth follicles, peripheral tissues, and oral mucosa (Nara, Dhanu, & Anandakrishna, 2010).

Herbal extracts such as garlic (Allium sativum) may pay more attention in the coming years as an alternative to NaOCl. A. sativum has a therapeutic effect via its broad spectrum antibacterial effect as well as its less cytotoxic effect (Khan et al., 2014; Prabhakaran & Mariswamy, 2018). However, a number of studies have been performed to assess A. sativum as an irrigant, especially in deciduous molars are limited. The aim of the trial is a clinical and radiographic assessment of garlic extract as an intracanal irrigant for pulpectomized primary molars in comparison with conventionally used NaOCl.

## 2 MATERIALS AND METHODS

### 2.1 Setting

The study was conducted on patients who attended outpatient clinic, Pediatric Dentistry department, Faculty of Dentistry, Minia University during the period from October 2016 to August 2018.

### 2.2 Sample size determination

With respect to the dichotomous variable, the sample size per group was calculated according to the following equation: *n* = 2(Zα/2 + Zβ)2P(1‐P)/(P1‐P2)2 (Charan & Biswas, 2013), where *n* is the number of subjects per group, P1 is success rate in the control group, P2 is the success rate in the experimental group, and P is the pooled prevalence and equals the prevalence in the experimental group (P1) subtracted from the prevalence in the control group (P2). The level of significance for the current study was adjusted at ≤0.05, and the power was 0.8, (Zα/2 = 1.65 and Zβ = 0.84). Up to the available data at the time of study conduction, there were no previous studies to estimate the success rate of garlic extract. Therefore, a pilot study was carried out, in which 12 infected primary molars were included and success rate of A. sativum irrigation was about 75%. The success rate prevalence of the control group was adjusted at 93%, which adopted from a previous study used the similar concentration of NaOCl for the current study (Trairatvorakul & Chunlasikaiwan, 2008). Thus, 52 molars in each group were suitable to provide 95% confidence interval (CI), and three more subjects per group were added to compensate the subject's attrition during the follow‐up period. Finally, the calculated sample size was 110 teeth (55 per group). The results of pilot study were not included into the analysis of the final results.

### 2.3 Study subjects

The total number of children enrolled in this investigation and applicable for specifications was 90 with 110 teeth. Their age ranged from 4 to 6 years.

### 2.4 Inclusion specifications

Clinical characteristics (Dentistry AAoP, 2009):

1. Class I or II according to American Society of Anesthesiologists (ASA)
2. Necrotic pulp tissues which may be asymptomatic or manifested with dull ache pain
3. Pathological tooth mobility
4. Percussion sensitivity
5. Swelling close to involved tooth accompanied with or without fistula

Radiographic characteristics of the root and supporting structures (Arikan, Sonmez, & Sari, 2016; Pandranki, NR, & Chandrabhatla, 2018):

1. The extension of radiolucency at the furcation area did not exceed the half of the space between the furcation and the permanent successor
2. No internal root resorption
3. External root resorption (Physiologic or pathologic) limited to apical third and with at least two‐thirds root intact

### 2.5 Exclusion specifications

1. Uncooperativeness of child and/or parents or caregiver's behavior
2. Unrestorable tooth
3. Presence of internal root resorption
4. Presence of calcific metamorphosis inside root canals
5. Presence of root resorption exceeding one third of its length

### 2.6 Randomization and allocation

Ninety eligible children have been randomly included in the study using computer generated block randomization list. Allocation was performed using properly sealed opaque envelope with treatment code and delivered by a resident in pediatric dentistry who completely ignorant of randomization code. The nature of irrigant solutions was masked for children and their parents/caregivers (i.e., single blinding).

### 2.7 Pulpectomy procedures

One‐stage pulpectomy was adopted in this study for treating primary molars without acute symptoms such as cellulitis or active discharge (Duggal, Nooh, & High, 2002). First, local anesthetic mepivicaine hydrochloride with levonordefrin 120,000 was administrated (ALEXANDERIA Co. for PHARMACUTICS, Egypt) and rubber dam application. Then, decay was removed, access to the pulp chamber and removal of the pulp chamber roof by # 558 non end cutting bur under air/water coolant. Coronal pulp tissue remnants were removed with sharp, sterile excavator, or large bur in a low speed handpiece. K‐files used for instrumentation of manually up to a # 30 to 35 (Goerig & Camp, 1983). According to the irrigant solution, the children assigned with 1:1 allocation ratio into two parallel groups: group (1) “experimental group,” 55 infected primary molars were treated with garlic extract, and group (2) “control group,” 55 infected primary molars were treated conventionally with 2.5 ml of 2.5% NaOCl every time the file was changed.

Then teeth in both groups flushed with 5 ml of 17% ethylenediamine tetraacetic acid (EDTA; PREVESTDenPro®, India) for 30 s as a chelating agent used for the removal of the inorganic portion of the smear layer (Mello, Kammerer, Yoshimoto, Macedo, & Antoniazzi, 2010). Finally, the root canal was rinsed with 5 ml of saline, then obturated with ZOE (PREVESTDenPro®, India), which mixed to medium consistency and delivered using lentulo spirals (MANI Inc.) and restored with a suitable restoration (i.e., Amalgam restorations for one surface endodontically treated molars and stainless steel crowns for more than one surface involved; Ibricevic & Al‐Jame, 2003). All cases recalled at 3, 6, and 12 months for clinical and radiographic evaluation.

### 2.8 Clinical and radiographic assessment

The assessment was implemented by two pediatric dentistry specialists, and the nature of the treatment was masked for both. The primary outcomes were to evaluate the efficiency of garlic extract as an irrigant of infected primary molars clinically and radiographically. The clinical criteria were scored according to the presence or absence of the following: (a) complain of pain, (b) swelling of gingiva, (c) fistulous tract, or (d) abnormal tooth mobility detected (Farooq, Coll, Kuwabara, & Shelton, 2000). The criteria of radiographic successes recorded at the base of existence of (a) root resorption, (b) persistent radiolucency at the furcation area up to 6 to 12 months after procedures, or (c) increase of the periapical and/or furcational radiolucency after treatment (Dentistry AAoP, 2009). Radiographic and clinical evaluation was scored independently. The presence of any negative sign, the tooth scored (0) and absence of all these abnormalities scored (1). The secondary outcomes were to detect the percent of different failure types accompanied by pulpectomy procedures after using garlic as an irrigant solution.

### 2.9 A. sativum extract preparation

A 100 g of garlic cloves has been cleaned, peeled, and dried. Ethanol of 70% concentration was added for 60 s. The cloves were placed in a laminar air flow chamber for evaporation of residual ethanol. Using a sterile mortar and pestle, cloves were homogenized aseptically and filtered through a double layer paper. The fully concentrated and extracted was diluted to the concentration of 25% with distilled water (Eswar, Venkateshbabu, Rajeswari, & Kandaswamy, 2013; Prabhakaran & Mariswamy, 2018).

### 2.10 Statistical testing

Data were analyzed using the intention to treat analysis. Statistical analysis performed using the Qui‐square test for binary variable. The level of significance was ˂0.05.

## 3 RESULTS

Out of 137 patients, 90 children with 110 teeth were selected for the current study. Their age ranged from 4 to 6 years, with a mean (±*SD*) of 4.7 ± 7.1 years. The majority of children were females represented 61.8%. Also, pulpectomy procedures were indicated in mandibular primary molars (61.8%) more frequently than the maxillary ones (38.2%). The mandibular second and first primary molars accounted for 33.6% and 28.2%, respectively, followed by the maxillary second primary molars (21.8%) and finally, the maxillary first primary molars (16.4%). All demographic data were demonstrated in Table 1.

**Table 1:** Distribution of demographic variables of participants

Demographic variables	Group (1) n (%)	Group (2) n (%)	Total n (%)
Gender			
Male	19 (42.2)	15 (33.3)	34 (37.8)
Female	26 (57.8)	30 (66.7)	56 (62.2)
Total	45 (50)	45 (50)	90 (100)
Age (years) Mean ± *SD*	4.8 ± 6.5	4.6 ± 7.7	4.7 ± 7.1
Teeth			
Maxillary			
First primary molars	9 (16.4)	9 (16.4)	18 (16.4)
Second primary molars	13 (23.6)	11 (20)	24 (21.8)
Mandibular			
First primary molars	14 (25.5)	17 (30.9)	31 (28.2)
Second primary molars	19 (34.5)	18 (32.7)	37 (33.6)
Total	55 (50)	55 (50)	110 (100)

Abbreviation: SD, standard deviation.

The interrater agreement for categorical items was measured using Cohen's kappa coefficient (*κ* = 0.94). This value indicated a strong agreement between the two examiners.

### 3.1 Clinical and radiographic success rates

**Figure 1:**
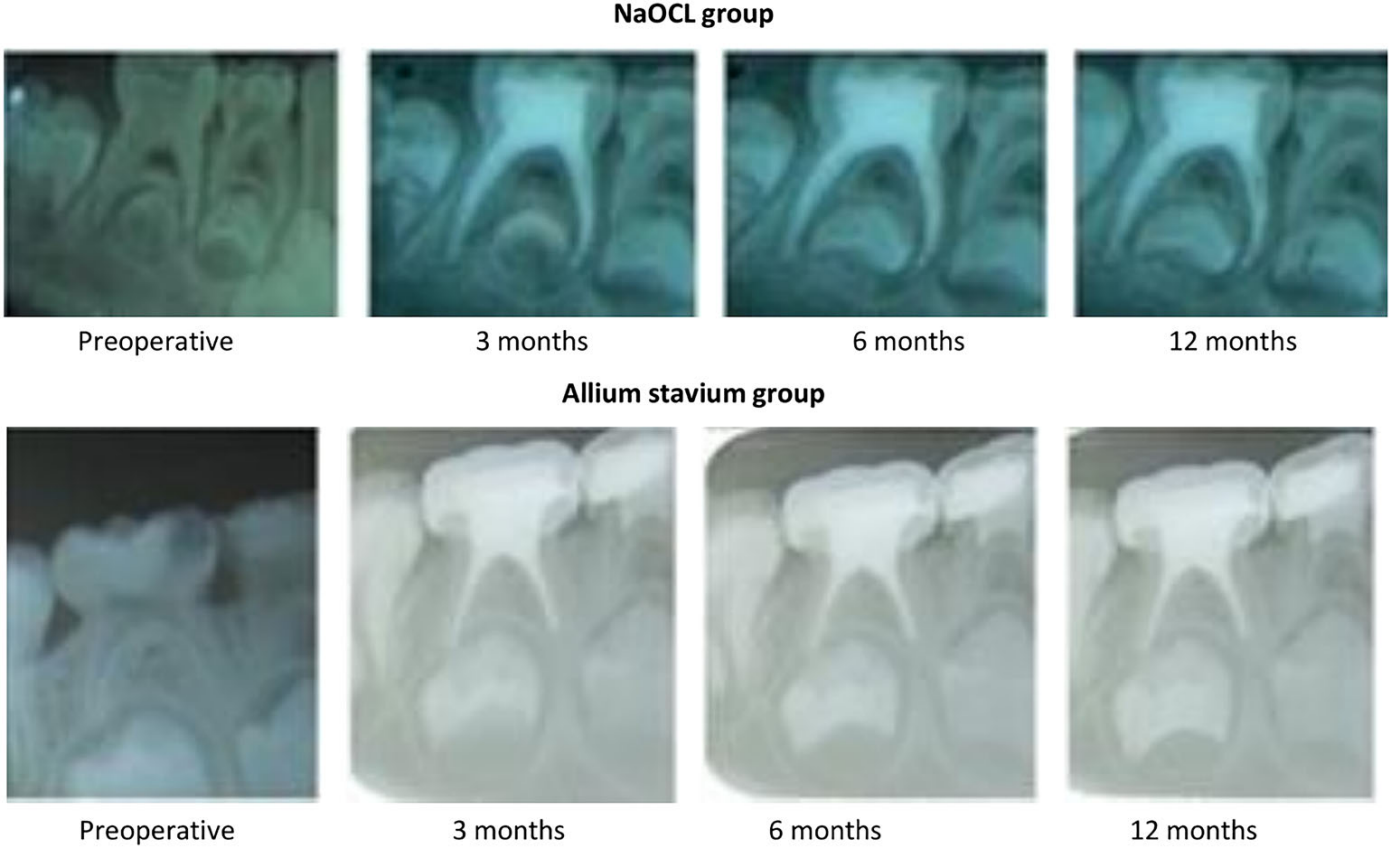
Periapical radiographs show successful pulpectomy of Allium sativum extract and sodium hypochlorite in the lower right second primary molars

All patients were available over the follow‐up period. After 3 months, all patients in both groups were available for evaluation (100%). The clinical success rates of A. sativum extract were 80%, whereas NaOCl irrigant showed 88.5%. Radiographic assessment reveals 72.7% and 89.1% success rates for the test and control group respectively. At 6 and 12 months, the clinical success rates of primary molar irrigated with A. sativum extract were similar 76.2%. On the other hand, the infected primary molars irrigated with NaOCl showed 87.3% and 89.1% clinical success at 6 and 12 months, respectively. Radiographic success rates of the A. sativum extract improved from 72.7% in 3 months to 74.5% and 76.4% at 6 and 12 months, respectively (Figure 1). For the control group, the recorded radiographic success rates were 85.5% at 3 months and 87.3% at 6 and 12 months. A statistically significant difference between the two groups could not be clinically or radiographically detected throughout the follow‐up period (*p* ˃ .05; Table 2).

**Table 2:** Clinical and radiographic success and failure rates at 3, 6, and 12 months

Follow‐up period	Group (1)	Group (2)	Qui‐square test (χ ^2^)
Clinical success rate at 3 months Clinical failure rate at 3 months	44 (80) 11 (20)	48 (87.3) 7 (12.7)	.30
Clinical success rate at 6 months Clinical failure rate at 6 months	42 (76.4) 13 (23.6)	48 (87.3) 7 (12.7)	.14
Clinical success rate at 12 months Clinical failure rate at 12 months	42 (76.4) 13 (23.6)	49 (89.1) 6 (10.9)	.08
Radiographic success rate at 3 months Radiographic failure rate at 3 months	40 (72.7) 15 (27.3)	47 (85.5) 7 (14.5)	.10
Radiographic success rate at 6 months Radiographic failure rate at 6 months	41 (74.5) 14 (25.5)	48 (87.3) 7 (12.7)	.09
Radiographic success rate at 12 months Radiographic failure rate at 12 months	41 (74.5) 14 (25.5)	48 (87.3) 7 (12.7)	.09

### 3.2 Clinical failure types

The common failures among children treated with garlic extract were tooth mobility accounted for 23.6%, followed by gingival swelling (20%), fistulous tract formation (18.2%), and finally, pain experience (12.7%). On the other side, tooth mobility recorded in 12.7% of infected molars treated with NaOCl, then gingival swelling and fistulous tract formation demonstrated 10.9%, and finally, 7.3% of the children complained of pain. In terms of the types of clinical failure, no statistically significant difference between the two groups (*p* ˃ .05; Table 3).

**Table 3:** Different types of clinical and radiographic failures

Failure type	Group (1)		Group (2)		Qui‐square test (χ ^2^)
	Present n (%)	Absent n (%)	Present n (%)	Absent n (%)	
Clinical failures					
Pain	7 (12.7)	48 (87.3)	4 (7.3)	51 (92.7)	.34
Gingival swelling	11 (20)	44 (80)	6 (10.9)	49 (89.1)	.19
Fistulous tract	10 (18.2)	45 (81.8)	6 (10.9)	49 (89.1)	.28
Tooth mobility	13 (23.6)	42 (76.4)	7 (12.7)	48 (87.3)	.14
Radiographic failures					
Persistent radiolucency	9 (16.4)	46 (83.6)	4 (7.3)	51 (92.7)	.14
Increase radiolucency	8 (14.5)	47 (85.5)	4 (7.3)	51 (92.7)	.22
Root resorption	14 (25.5)	41 (74.5)	7 (12.7)	48 (87.3)	.09

### 3.3 Radiographic failure types

In regard to, persistent radiolucency, the increase in periapical and/furcational radiolucency and root resorption, there was no statistically significant difference between the two groups (*p* ˃ .05; Table 3).

## 4 DISCUSSION

The integration between root canal mechanical cleaning and shaping, irrigation, and filling is essential for successful pulpectomy process (Kandaswamy & Venkateshbabu, 2010). Up to the available data, the use A. sativum extract in endodontic treatment of primary teeth is limited especially the in vivo studies. Thus, the current study was conducted to compare garlic extract as an intracanal irrigant for pulpectomized primary molars with conventionally used NaOCl. Irrigation aimed to help in expel of pulp tissue, debris, and pathogenic microorganisms (Zehnder, 2006). NaOCl action is dual via (a) its oxidizing capability on microorganisms and (b) distortion of dentin collagen structure causing dissolution (Kandaswamy & Venkateshbabu, 2010). The other arm of this study was garlic extract showed has antibacterial properties that have been proved against some microorganisms such as *Pseudomonas*, *Klebsiella*, *Streptococcus mutans*, and *Porphyromonas gingivalis*. Also, it was reported to be effective against *E. faecalis* similar to autoclave (Hugar et al., 2017). Garlic extract has better antibacterial properties when compared with calcium hydroxide (Eswar et al., 2013). The antibacterial characteristics of garlic attributed to one of its active components called thiosulfinates (e.g., Allicin; Ankri & Mirelman, 1999). The antimicrobial properties of allicin is chiefly attributed the total inhibition of RNA synthesis and partial inhibition of DNA and protein syntheses, suggesting that RNA is the primary target of allicin action (Feldberg et al., 1988).

### 4.1 Clinical and radiographic success

The clinical and radiographic success rates of pulpectomy in primary teeth demonstrate diversity among different studies. This variation might be attributed to the differences in study design in terms of; (a) inclusion and exclusion criteria; (b) follow‐up periods; (c) pulpectomy technique, including mechanical instrumentation, irrigants and their concentration, and the filling material used for obturation; and (d) pulpectomy method (i.e., one versus two visits method). All of these variables have to be taken into consideration when comparing the current study results with other studies. However, it is useful to mention some of studies for comparison. The clinical result of the control group in the current study was 89.1% after 12 months, which slightly lower than the results of a study performed by Trairatvorakul and Chunlasikaiwan. They reported 96% and 93% clinical success rates at 6 and 12 months, respectively, out of 27 infected primary molars irrigated using 2.5% NaOCl. Although the radiographic success rate was 85% after 12 months of the follow‐up period, which was comparable with this study results (Trairatvorakul & Chunlasikaiwan, 2008). Chen et al. (2017) reported 100% clinical and radiographic success rates at 6 and 12 months success rates of ZOE pulpectomy (Chen, Liu, & Zhong, 2017). These differences in findings might be related to the use of two visits pulpectomy method. Coll et al. (1985) used NaOCl in irrigation of 41 infected primary molars, and the success was 33 molars represented 80.5% in the first posttreatment revaluation in 6 to 36 months (mean 21 months), which considered to some extent comparable with the results of both groups in the current study (Coll, Josell, & Casper, 1985). The clinical and radiographic success varies from 85% 14 to 100% in a ZOE group of one‐visit pulpectomy for 20 infected primary molars (Barcelos, Santos, Primo, Luiz, & Maia, 2011). This difference could be explained by the smaller the used sample size than that used in the present study. The findings of children of age 37 months or more revealed a 74.4% success for posterior teeth in a study performed by Coll and Sadrian (1996), and this is consistent with the current study results of the experimental group (Coll & Sadrian, 1996). Mortazavi and Mesbahi (2004) reported a 78.5% overall success rate of 52 necrotic primary teeth pulpectomized with ZOE (Mortazavi & Mesbahi, 2004). Mani et al. published an 83.3% clinical and radiographic success of ZOE pulpectomy when compared with calcium hydroxide pulpectomy in 60 cases (Ingle, 2008). Another study compared clinical and radiographic success rates of endoflas to ZOE using 3% NaOCl, the clinical success radiographic success after 12 months follow‐up period were 89% and 63%, respectively (Pandranki et al., 2018).

### 4.2 Types of clinical and radiographic failures

In the current study, abnormal tooth mobility was the dominantly observed clinical failures and periapical and/or furcational radiolucency after treatment. This is in agreement with Trairatvorakul and Chunlasikaiwan who reported 11% with pathological mobility and 22% of pulpectoized teeth revealed severe radiographic pathology (Trairatvorakul & Chunlasikaiwan, 2008). Also, the pathologic external root resorption and/or apical radiolucency were identified as the common radiographic failures in a study performed by Primosch, Ahmadi, Setzer, and Guelmann (2005).

The limitations of the current study were the strict specification criteria during subject selection stage and the relatively limited duration of the follow‐up. Another restriction was the nature of treatment which could not be masked to the operator (single blinded study). However, the two specialists who performed clinical and radiographic assessments were blinded to the nature of treatment. Another shortage of the current study is the relatively short period of follow‐up.

On the other hand, the current study owns some merits in terms of the following: (a) it is one of the in vivo randomized clinical trials which evaluate an herbal substance such as A. sativum extract as irrigant of infected root canals of primary molars, (b) the number of enrolled subjects was adequate, and finally, (c) this study may provide a reasonable scientific background for further researches in this era with more included subjects and longer follow‐up interval.

## 5 CONCLUSIONS

There is no statistically significant difference between A. sativum extract as an irrigant and sodium hypochlorite 12 months follow‐up period. Garlic extract provides a good natural and potent antibacterial agent that can be used safely for irrigation of root canals of primary molars.

## DECLARATIONS

Ethics approval and consent to participate the current research has been conducted in full accordance with the World Medical Association Declaration of Helsinki. The trial registration number on ClinicalTrials.govPRS (Protocol registration and Result System) is NCT03795636. Permission from the ethic committee of Faculty of Dentistry, Minia University, was obtained. Each potential subject must be adequately informed of the aims, methods, sources of funding, any possible conflicts of interest, institutional affiliations of the researcher, the anticipated benefits and potential risks of the study and the discomfort it may entail, and any other relevant aspects of the study. The study can be conducted only after ensuring that the potential subject has understood the information and obtaining a freely given written informed consent signed by parents or legal guardians of study participants. Also, parents/caregivers signed for publishing the results of the study. Teeth recorded as a failure were retreated if indicated. Although hopeless teeth were extracted and space maintainers were constructed if required.

## CONFLICT OF INTEREST

None declared.
